# Supplementary material for: Hybrid Models and Biological Model Reduction with PyDSTool
Source: PLoS Comput Biol. 2012 Aug 9;8(8):e1002628. doi: 10.1371/journal.pcbi.1002628 (PMC3415397; doi:10.1371/journal.pcbi.1002628)
Supplement: Text S4 — Complete source code for the PyDSTool package (version 0.88.120504). Includes API documentation and help files linking to web pages. This file is identical to the current public release on Sourceforge.net. (ZIP) [file pcbi.1002628.s004.zip › PyDSTool/html/PyDSTool.Toolbox.optimizers.step.goldfeld_step-pysrc.html]

xml version="1.0" encoding="ascii"?


PyDSTool.Toolbox.optimizers.step.goldfeld\_step


| Home | Trees | Indices | Help | PyDSTool |
| --- | --- | --- | --- | --- |

Package PyDSTool ::
Package Toolbox ::
Package optimizers ::
Package step ::
Module goldfeld\_step

[hide private]

[frames] | no frames]

# Source Code for Module PyDSTool.Toolbox.optimizers.step.goldfeld\_step

```
 1   
 2  """ 
 3  Computes Goldfeld step for a specific function at a specific point 
 4  """ 
 5  from numpy import pi,  dot, cos 
 6  from numpy.linalg import solve as n_solve 
 7  from numpy.linalg import norm, cholesky, eigvalsh 
 8   


9 -class GoldfeldStep(object):


10       
11    """ 
12    The Goldfeld step 
13    """ 


14 -  def __call__(self, function, point, state):


15      """ 
16      Computes Goldfeld step  
17      """ 
18      g = function.gradient(point) 
19      state['gradient'] = g 
20      G = function.hessian(point) 
21      state['hessian'] = G 
22      c = 1e-8 # is this one best? 
23       
24       
25       
26      d0 = None 
27       
28      try: 
29          L = cholesky(G) 
30          # reach here => isPositiveDefinite = True 
31          step = n_solve(L.T, n_solve(L, -g)) 
32      except: 
33          # isPositiveDefinite = False 
34          G_eigvals = eigvalsh(G) 
35          minEig = min(G_eigvals) 
36          if minEig < 0: 
37              shift = -minEig + c 
38               
39              #avoiding sparse case with big nVars 
40              for i in xrange(point):  G[i,i] += shift 
41                   
42          step = n_solve(G, -g) 
43    
44       
45      state['direction'] = step 
46      return step

47
```

  


| Home | Trees | Indices | Help | PyDSTool |
| --- | --- | --- | --- | --- |

|  |  |
| --- | --- |
| Generated by Epydoc 3.0.1 on Fri May 4 15:24:26 2012 | http://epydoc.sourceforge.net |
